# *miR-489* is a tumour-suppressive miRNA target *PTPN11* in hypopharyngeal squamous cell carcinoma (HSCC)

**DOI:** 10.1038/sj.bjc.6605811

**Published:** 2010-08-10

**Authors:** N Kikkawa, T Hanazawa, L Fujimura, N Nohata, H Suzuki, H Chazono, D Sakurai, S Horiguchi, Y Okamoto, N Seki

**Affiliations:** 1 Department of Functional Genomics, Graduate School of Medicine, Chiba University, Chiba, Japan; 2 Department of Otorhinolaryngology/Head and Neck Surgery, Graduate School of Medicine, Chiba University, Chiba, Japan; 3 Biomedical Research Center, Chiba University, Chiba, Japan

**Keywords:** hypopharyngeal squamous cell carcinoma (HSCC), microRNA, *miR-489*, *PTPN11*

## Abstract

**Background:**

Hypopharyngeal squamous cell carcinoma (HSCC) is an aggressive malignancy with one of the worst prognoses among all head and neck cancers. Greater understanding of the pertinent molecular oncogenic pathways could help improve diagnosis, therapy, and prevention of this disease. The aim of this study was to identify tumour-suppressive microRNAs (miRNAs), based on miRNA expression signatures from clinical HSCC specimens, and to predict their biological target genes.

**Methods:**

Expression levels of 365 human mature miRNAs from 10 HSCC clinical samples were screened using stem-loop real-time quantitative PCR. Downregulated miRNAs were used in cell proliferation assays to identify a tumour-suppressive miRNA. Genome-wide gene expression analyses were then performed to identify the target genes of the tumour-suppressive miRNA.

**Results:**

Expression analysis identified 11 upregulated and 31 downregulated miRNAs. Gain-of-function analysis of the downregulated miRNAs revealed that *miR-489* inhibited cell growth in all head and neck cancer cell lines examined. The gene *PTPN11* coding for a cytoplasmic protein tyrosine phosphatase containing two Src Homology 2 domains was identified as a *miR-489*-targeted gene. Knockdown of *PTPN11* resulted in the inhibition of cell proliferation in head and neck SCC cells.

**Conclusion:**

Identification of the tumour-suppressive miRNA *miR-489* and its target, *PTPN11*, might provide new insights into the underlying molecular mechanisms of HSCC.

Hypopharyngeal squamous cell carcinoma (HSCC) is a relatively rare disease, with an incidence of about 10 cases per million people-years (Davies and Welch, 2006). Hypopharyngeal squamous cell carcinoma has a very poor prognosis compared with other head and neck squamous cell carcinomas (HNSCCs), with 5-year survival rates ranging from 30 to 35% (Hoffman *et al*, 1997; Bova *et al*, 2005). This poor prognosis is thought to result from advanced primary disease, a high rate of loco-regional recurrence, distant metastasis, and second primary tumours (Spector *et al*, 2001; Helliwell, 2003). Survival rates of HSCC patients have not markedly improved despite recent advances in various treatment modalities, including surgery, radiotherapy, and chemotherapy (Godballe *et al*, 2002). Understanding the molecular oncogenic pathways underlying HSCC could significantly improve diagnosis, therapy, and prevention of the disease.

MicroRNAs (miRNAs) are endogenous small non-coding RNAs that can control gene expression by targeting messenger RNAs (mRNAs) for cleavage or translational repression (Bartel, 2004). The miRNAs are involved in crucial biological processes, including development, differentiation, apoptosis, and proliferation (Bartel, 2004; Kloosterman and Plasterk, 2006). An important role for miRNAs in the development of cancer has emerged in recent years (Hwang and Mendell, 2006). The miRNAs are aberrantly expressed in many human cancers, and they may function as oncogenes and tumour suppressors. Upregulated miRNAs could function as oncogenes by negatively regulating tumour suppressor genes, while, downregulated miRNAs could act as tumour suppressors, inhibiting cancers by regulating oncogenes (Esquela-Kerscher and Slack, 2006; Hammond, 2006; Zhang *et al*, 2007).

A growing body of evidence indicates that unique miRNA expression profiles associated with particular cancers could serve as useful biomarkers for disease prognosis and diagnosis (Lu *et al*, 2005; Calin and Croce, 2006; Childs *et al*, 2009). Studies have been carried out for the purpose of identifying specific miRNA alterations in HNSCC (for review, see Liu *et al*, 2009). However, limited data are available on miRNA expression signatures in HSCC clinical specimens (Childs *et al*, 2009; Ramdas *et al*, 2009; Hui *et al*, 2010). Knockdown or overexpression of a specific miRNA allows functional investigation and validation of the specific role of the miRNAs in tumourigenesis. Analysis of the expression signature of laryngeal, oropharyngeal, or hypopharyngeal cancers showed that underexpression of *miR-375* and overexpression of *miR-106b-25* cluster might contribute to oncogenesis (Hui *et al*, 2010).

In this functional analysis of miRNA in HSCC, differentially expressed miRNAs were identified by evaluating 365 mature miRNAs from clinical specimens of HSCC. Cell proliferation assays were conducted to identify tumour-suppressive miRNAs, and genome-wide gene expression analysis was used to identify their targets. The identification of tumour-suppressive miRNAs, and their corresponding target genes, could provide new insights into HSCC carcinogenesis.

## Materials and Methods

### Clinical HSCC specimens

Tissue specimens of HSCC and adjacent non-cancerous hypopharynx tissue were obtained from patients undergoing surgical treatment for HSCC at Chiba University Hospital between 2004 and 2009. Tissues were immediately frozen in liquid nitrogen and stored at −80°C until further processing. Non-cancerous tissues were obtained far from the centre of the cancer in surgical specimens. No cancer cells were detected in neighbouring formalin-fixed paraffin-embedded specimens. Infection by human papillomavirus (HPV) types HPV16, HPV18, and HPV33 was investigated using genomic DNA from clinical specimens with the PCR Human Papillomavirus Detection Set (Takara, Tokyo, Japan) according to the manufacturer's instructions.

This study was approved by the Bioethics Committee of Chiba University. Prior written informed consent and approval were obtained from all patients.

### Cell lines and cell culture

Four of the squamous cell carcinoma cell lines (FaDu, HSC2, HSC3, and D562) were maintained in Dulbecco's Modified Eagle's Medium/Nutrient Mixture F-12 Ham (Invitrogen, Carlsbad, CA, USA), supplemented with 10% foetal bovine serum (Invitrogen) in a humidified atmosphere containing 5% CO_2_ at 37°C. The FaDu cell line was derived from HSCC tissue (Rangan, 1972). The three remaining cell lines were derived from oral floor (HSC2), tongue (HSC3), and nasopharynx (D562) (Peterson *et al*, 1971; Momose *et al*, 1989).

### RNA isolation

Total RNA was isolated using TRIzol reagent (Invitrogen) according to the manufacturer's protocol. The concentrations of RNA were determined using a spectrophotometer, and molecule integrity was checked by gel electrophoresis. The quality of RNA was confirmed using an Agilent 2100 Bioanalyzer (Agilent Technologies, Santa Clara, CA, USA).

### The miRNA expression signatures and data normalisation

The miRNA expression patterns were evaluated using the TaqMan Low Density Array Human MicroRNA Panel v1.0 (Applied Biosystems, Foster City, CA, USA). The assay was composed of two steps: generation of complementary DNA (cDNA) by reverse transcription, followed by a TaqMan real-time PCR assay. Briefly, miRNAs in the samples were converted to cDNA using 365 specific stem-loop reverse transcription primers. After cDNA conversion, the quantity of mature miRNAs was evaluated using specific TaqMan real-time PCR primers and probes. Real-time PCR was performed in duplicate using GeneAmp Fast PCR Master Mix (Applied Biosystems) and the ABI 7900HT Real-Time PCR System (Applied Biosystems). The *C*_t_ values were transformed using the following formula: expression score=2^(40−*C*t)^, and the calculated data were uploaded into GeneSpring GX version 7.3.1 software (Silicon Genetics, Redwood City, CA, USA) as described previously (Ichimi *et al*, 2009; Kano *et al*, 2010). Description of real-time PCR and the list of human miRNAs can be found on the Applied Biosystems website.

Three approaches were used to normalise the miRNA expression data: global normalisation and endogenous gene normalisation based on *RNU44* and *RNA48* (Ichimi *et al*, 2009; Kano *et al*, 2010). The miRNAs that were detected by all these three normalisation methods were chosen for further study. The fold change, normalisation ratio and *P*-values were calculated during global normalisation.

### Mature miRNA transfection

Mature miRNA molecules, pre-miR miRNA precursors, and a negative control (Applied Biosystems) were incubated with Opti-MEM (Invitrogen) and Lipofectamine RNAiMax reagent (Invitrogen) as described previously (Ichimi *et al*, 2009). Transfection efficiency of pre-miR in the cell lines was confirmed on the basis of downregulation of *PTK9* mRNA by transfection with *miR-1* (as recommended by Applied Biosystems).

### XTT (cell proliferation) assay

Cells were transfected with 10 nM miRNA by reverse transfection and plated into 96-well plates at 3 × 10^3^ cells per well. After 72 h, cell viability was determined with the XTT assay, using Cell Proliferation Kit II (Roche Molecular Biochemicals, Mannheim, Germany) as described previously (Kano *et al*, 2010). Triplicate wells were assayed for cell viability in each treatment group.

### Target gene search for miR-489

Expression profiles of FaDu cells transfected with *miR-489* were screened and compared against miRNA-negative control transfectants using Oligo-microarray Human 44K arrays (Agilent Technologies; Chiyomaru *et al*, 2010; Kano *et al*, 2010). Hybridisation and washing steps were performed as described previously (Sugimoto *et al*, 2009). The arrays were scanned using a Packard GSI Lumonics ScanArray 4000 (Perkin Elmer, Boston, MA, USA). The data were analysed using DNASIS array software (Hitachi Software Engineering, Tokyo, Japan), which converted the signal intensity of each spot into text. The log2 ratios of the median subtracted background intensity were analysed. Data from each microarray study were subjected to a global normalisation (Sugimoto *et al*, 2009).

The predicted target genes and their conserved miRNA-binding site seed regions were investigated using TargetScan (release 5.1, http://www.targetscan.org/). The sequences of the predicted mature miRNAs were confirmed using miRBase release 13.0 (http://microrna.sanger.ac.uk/).

### Real-time quantitative RT–PCR

First-strand cDNA was synthesised from 1 *μ*g total RNA using random primers and the Reverse Transcription (RT) System (Promega, Tokyo, Japan). Gene-specific PCR products were assayed continuously using a 7900-HT Real-Time PCR System with TaqMan probes and primers for *PTPN11* (P/N: Hs00818825_m1, Assay-On-Demand Gene Expression Products; Applied Biosystems), according to the manufacturer's protocol. The initial PCR step consisted of a 10-min hold at 95°C, followed by 40 cycles of 15-s denaturation at 95°C, and 1 min annealing/extension at 63°C. For cell lines and clinical samples, *GAPDH* (A/N: NM_002046) and *18S rRNA* (P/N: 4333760F), respectively, were used as internal controls (Assay-On-Demand Gene Expression Products; Applied Biosystems). All reactions were performed in triplicate, and included negative control reactions that lacked cDNA.

### Immunoblotting

Cells were collected 72 h after transfection and protein lysates were prepared. A total of 50 *μ*g of lysate was separated by NuPAGE on a 4–12% bis–tris gel (Invitrogen) and transferred onto a polyvinylidene fluoride membrane. Immunoblotting was performed with diluted (1 : 100) monoclonal anti-PTPN11 antibody (ab76285, Abcam, Cambridge, UK), with *β*-actin serving as an internal control. The membrane was washed and incubated with goat anti-mouse IgG (H+L)–HRP conjugate (Bio-Rad, Hercules, CA, USA). Specific complexes were visualised by echochemiluminescence (GE Healthcare Bio-Sciences, Princeton, NJ, USA).

### Plasmid construction and dual-luciferase assay

The *miR-489* target sequences were chemically synthesised (Takara, Tokyo, Japan) and inserted between the *Xho*I and *Pme*I restriction sites in the 3′ UTR of the *hRluc* gene in the psiCHECK-2 vector (Promega). FaDu cells were then transfected with 5 ng vector, 10 nM mature miRNA molecules, pre-miRNA *miR-489* (Applied Biosystems), and 1 *μ*g Lipofectamine 2000 (Invitrogen) in 100 *μ*l Opti-MEM. Firefly and *Renilla* luciferase activities in cell lysates were determined using a dual-luciferase assay system (Promega). Normalised data were calculated as the quotient of *Renilla*/firefly luciferase activities.

### Small interfering RNA treatment

After co-transfection of 1 or 10 nM small interfering RNA *PTPN11* (si-PTPN11; ID S11524, Ambion) or non-silencing small interfering RNA (si-control), FaDu cells were seeded into 96-well plates at a density of 3 × 10^3^ cells per well. After 72 h, cell viability was determined using the XTT assay. Triplicate wells were measured for cell viability in each treatment group.

### Statistical analysis

The relationships between two groups and the numerical values obtained by real-time RT–PCR were analysed using the non-parametric Mann–Whitney *U* test or the paired *t*-test. The relationship between *miR-489* expression and *PTPN11* expression was analysed using the Spearman rank correlation. Expert StatView (version 4, SAS Institute, Cary, NC, USA) was used for analyses, with statistical significance defined as *P*<0.05.

## Results

### Identification of differentially expressed miRNAs in clinical HSCC specimens

The expression of 365 mature miRNAs was evaluated in matched pairs of HSCC and their adjacent non-cancerous tissues from 10 patients (Table 1) after HPV infection was ruled out in all specimens. Following three normalisations (*RNU44*, *RNU48* and global) of the raw data, 42 differentially expressed miRNAs were found using all three methods. Of these, 11 (3.0%) were upregulatedd and 31 (8.5%) were downregulated in cancerous tissues. The fold change, normalisation ratio, and *P*-values in Tables 2A and B were revealed by global normalisation.

### Identification of tumour-suppressive miRNAs

**Figure 1:**
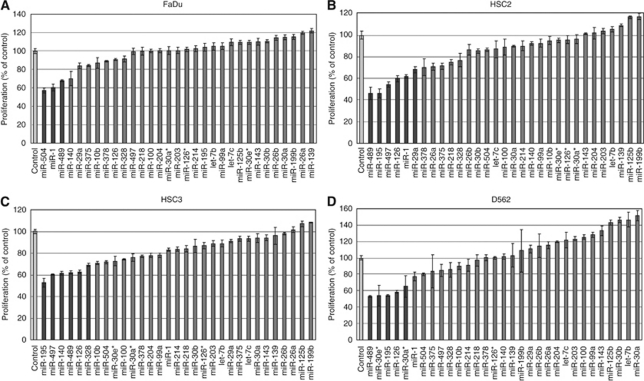
Effect of transfection with 31 downregulated miRNAs on cancer cell proliferation. Cancer cells were transfected with 10 nM of the indicated mature miRNA. After incubation for 72 h, cell proliferation was determined using XTT assays. (**A**) FaDu cells; (**B**) HSC2 cells; (**C**) HSC3 cells; (**D**) D562 cells. The darkly shaded bars represent a decrease in cell proliferation of more than 30% compared with control transfections.

The effect of increasing levels of downregulated miRNAs on cancer cell proliferation was used to identify miRNAs with tumour suppression activity. The proliferation rates of HSCC transfectants are shown in Figures 1A–D. ‘Cell growth inhibiting miRNAs’ were defined as miRNA species that decreased cell proliferation by more than 30% compared with control transfectants. Three miRNAs (*miR-504*, *miR-1*, and *miR-489*) showed cell growth inhibition in FaDu cells (Figure 1A). Similarly, six miRNAs (*miR-489*, *miR-195*, *miR-497*, *miR-126*, *miR-1*, and *miR-29a*) were identified in HSC2 cells (Figure 1B), six miRNAs (*miR-195*, miR*-497*, *miR-140*, *miR-489*, *miR-126,* and *miR-328*) in HSC3 cells (Figure 1C), and five miRNAs (*miR-489*, miR-30e*, *miR-195*, *miR-126,* and miR-30a*) in D562 cells (Figure 1D). Of the 31 downregulated miRNAs (Table 2B), *miR-489* inhibited cell growth in all four of the cancer cell lines tested and was, therefore, chosen for further study.

### Screening of miR-489 target genes by genome-wide gene expression analysis

The molecular basis of *miR-489* tumour suppression in HSCC was investigated by examining the effect of *miR-489* on protein-coding genes. Mature *miR-489* was transiently transfected into FaDu cells, with negative-miRNA transfection used as a control. Comprehensive gene expression analysis showed changes in gene expression patterns between *miR-489* and negative-control transfectants. To identify candidate *miR-489* target genes, a cut-off of values less than −2.00-fold was applied to the array data. This filtering resulted in the detection of 53 genes that were significantly downregulated upon *miR-489* transfection (Table 3). Entries from the microarray data were approved by the Gene Expression Omnibus, and were assigned the Gene Expression Omnibus accession number GSE19718.

The 3′ UTR regions of these downregulated genes were examined for *miR-489* target sites using the TargetScan database. Of the 53 putative gene targets, 32 genes contained *miR-489* target sites (Table 3).

### Effect of miR-489 transfection on PTPN11 expression in cancer cells

One of the genes with *miR-489* target sites in its 3′ UTR is *PTPN11*. This gene encodes a protein tyrosine phosphatase (PTP) that contains two Src Homology 2 domains. Although PTPs generally act as tumour suppressors, *PTPN11* has been identified as the first PTP oncogene (Tonks, 2006). Therefore, this gene was investigated further as a target of *miR-489*.

**Figure 2:**
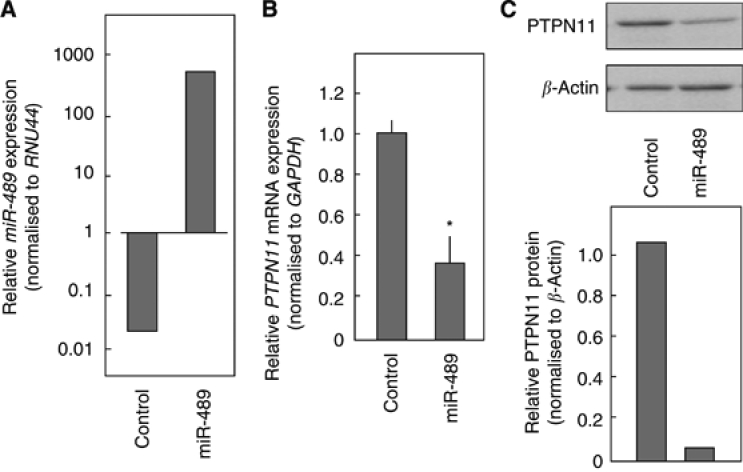
*miR-489* negatively regulates *PTPN11* expression. FaDu cells were transfected with *miR-489.* After incubation for 72 h, total RNA and proteins were isolated. (**A**) FaDu cells were treated with a miR-negative control (10 nM) or miR-489 (10 nM). After 72 h, *miR-489* expression was measured by TaqMan quantitative real-time PCR. The results are normalised to *RNU44* expression. (**B**) *PTPN11* mRNA expression was analysed by TaqMan quantitative real-time PCR. The results are normalised to *GAPDH* expression and are presented relative to control expression. ^*^ *P*<0.05. (**C**) Cell lysates were analysed by immunoblotting. Membranes were incubated with anti-PTPN11 IgG and anti-*β*-actin IgG. The autoradiographic density of each protein band was quantified using NIH ImageJ software. The results are standardised against *β*-actin levels and are presented as the relative density.

To determine whether *miR-489* regulates *PTPN11* expression, *miR-489* was introduced into FaDu cells. Gain-of-function effects of miR-489 were investigated 72 h after transfection. The expression of miR-489 was elevated by >1000-fold in FaDu cells compared with the miR-negative control (Figure 2A). The mRNA levels for *PTPN11* were significantly repressed (Figure 2B). Immunoblotting confirmed that PTPN11 protein expression was significantly decreased in *miR-489* transfectants (Figure 2C).

**Figure 3:**
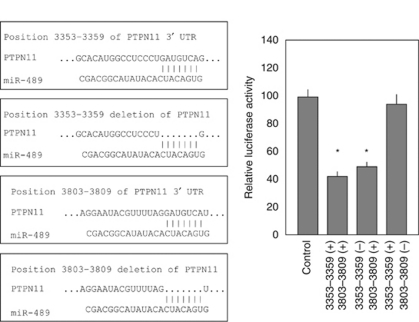
*miR-489* binds to the 3′ UTR of *PTPN11* mRNA. A luciferase reporter assay used a vector encoding the partial *PTPN11* 3′ UTR (position 3300–3850). *Renilla* luciferase values are normalised against firefly luciferase values. Luciferase reporter assays were repeated using mutated vectors in which the candidate sites targeted by the *miR-489* were deleted. ^*^P<0.05

A luciferase reporter assay was performed to determine whether *PTPN11* mRNA contains a *miR-489* target site, as predicted by the TargetScan algorithm. A vector encoding the partial 3′ UTR of *PTPN11* (position 3300–3850) exhibited significantly decreased luminescence intensity after *miR-489* transfection (Figure 3). To determine the specific site targeted by *miR-489*, two vectors carrying deletions of candidate target sites were constructed (deleted positions 3353–3359 and 3803–3809). Luminescence intensity was significantly decreased for the vectors carrying the 3′ UTR and the deletion at position 3353–3359, but not in the vector with the deletion at position 3803–3809 (Figure 3), indicating that the region between positions 3803–3809 contains the *miR-489* target site.

### Effect of si-PTPN11 transfection

**Figure 4:**
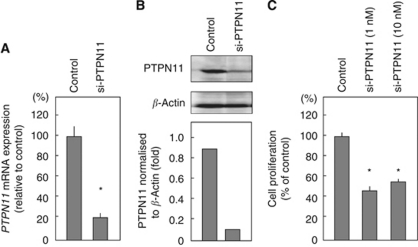
Proliferation is inhibited by transfection with *si-PTPN11* in FaDu cells. FaDu cells were transfected with 10 nM *si-PTPN11.* Total RNA and proteins were isolated after 72-h incubation. (**A**) *PTPN11* mRNA expression was analysed by TaqMan quantitative real-time PCR. The results are normalised to *GAPDH* expression and are presented as relative to control expression. ^*^P<0.05. (**B**) Cell lysates were analysed by immunoblotting. Membranes were incubated with anti-PTPN11 IgG and anti-*β*-actin IgG. The autoradiographic density of each protein band was quantified using NIH ImageJ software. The results are standardised against *β*-actin levels and are presented the relative density. (**C**) FaDu cells were transfected with 1 or 10 nM si-PTPN11. After incubating for 72 h, cell proliferation was determined using an XTT assay. ^*^P<0.05.

A loss-of-function assay using small interfering RNA analysis was performed to examine the oncogenic function of *PTPN11*, which is directly targeted by *miR-489*. The effect of si-PTPN11 on mRNA and protein expression levels was evaluated after transfection into FaDu cells. Both PTPN11 mRNA and protein levels had been reduced 72 h after transfection (Figures 4A and B). The contribution of PTPN11 to cell viability was assessed with si-PTPN11 loss-of-function assays in FaDu cells. Knockdown of *PTPN11* significantly decreased cancer cell growth compared with si-control transfectants (Figure 4C).

### PTPN11 overexpression in HSCC clinical specimens

**Figure 5:**
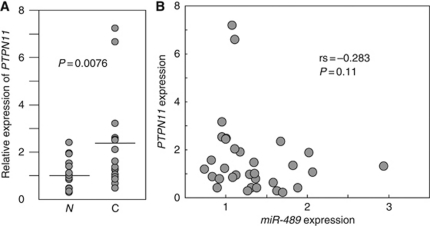
*PTPN11* overexpression in clinical HSCC specimens. (**A**) *PTPN11* mRNA expression levels were analysed by TaqMan quantitative real-time PCR and normalised to *18S rRNA* expression. *PTPN11* mRNA expression was compared between matched HSCC and non-cancerous tissues in 16 patients. Data were analysed using the paired *t*-test. N, non-cancerous tissues; C, cancer tissues. (**B**) Correlation between *PTPN11* and *miR-489* expression in HSCC clinical specimens.

The mRNA expression levels of *PTPN11* were significantly higher in 16 HSCC tissues than in adjacent non-cancerous hypopharyngeal tissues (Figure 5A). The possibility that the expression of *PTPN11* and the *miR-489* were correlated was tested using the Spearman rank correlation. However, the inverse correlation between *PTPN11* and *miR-489* expression levels was too low to be statistically significant (*rs*=−0.283 and *P*=0.11; Figure 5B).

## Discussion

Unique miRNA expression profiles associated with particular cancers could serve as biomarkers for prognosis and diagnosis (Lu *et al*, 2005; Calin and Croce, 2006; Childs *et al*, 2009). This study of miRNA expression signatures in clinical HSCC specimens resulted in the identification of 42 differentially expressed miRNAs, of which 11 were upregulatedd (Table 2A) and 31 were downregulated (Table 2B). As HSCC has a very poor prognosis compared with other HNSCCs, these HSCC miRNA expression signatures could help elucidate the underlying molecular mechanisms of this disease.

The miRNA expression signatures of head and neck cancers have been reported by several laboratories (Chang *et al*, 2008; Wong *et al*, 2008; Avissar *et al*, 2009; Chen *et al*, 2009; Childs *et al*, 2009). A comparison of our data with these published expression signatures revealed that *miR-21*, *miR-18a*, and *miR-196b* are commonly upregulatedd in head and neck cancers. It was already known that *miR-21*, which functions as an oncogene (Chang *et al*, 2008), stands out as the miRNA most often overexpressed across a diverse range of malignancies (Esquela-Kerscher and Slack, 2006). Further studies are needed to clarify the functions of these upregulatedd miRNAs and their role in HSCC carcinogenesis.

A total of 17 of the 31 downregulated miRNAs identified in this study (miR-1, miR-375, miR-139–5p, miR-125b, miR-199b, miR-100, miR-497, miR-30a, miR-218, miR-10b, miR-204, miR-143, miR-99a, miR-195, miR-140–5p, miR-26b, and miR-30b) are previously reported head and neck cancer signatures. In HNSCC, miR-125b and miR-100 have tumour-suppressive functions (Henson *et al*, 2009). The miR-375 was the most downregulated miRNA in the HNSCC samples, including hypopharyngeal cancer, and its increased expression leads to a significant reduction in cell viability in cancer cells (Hui *et al*, 2010).

Tumour-suppressive miRNAs are usually underexpressed in cancer cells (Esquela-Kerscher and Slack, 2006; Hammond, 2006; Zhang *et al*, 2007). Therefore, we hypothesised that miRNAs with HSCC tumour-suppressive activity could be among the 31 downregulated miRNAs. In a screen for miRNAs that inhibited cancer cell proliferation, *miR-489* inhibited cell growth in all cell lines examined (Figure 1), and was identified as a tumour-suppressive miRNA in HSCC. Although little is currently known regarding the function of *miR-489*, a recent report indicated that *miR-489* may regulate early osteogenic differentiation in human mesenchymal stem cells, and that *miR-489* has critical roles in osteogenesis (Schoolmeesters *et al*, 2009). However, the relationship between *miR-489* and carcinogenesis remains unclear.

As miRNAs function by negatively regulating protein-coding genes, it is important to understand the miRNA-target gene network. Potential targets of *miR-489* were observed in a genome-wide screen using FaDu (HSCC) cells. Of the 53 candidate genes, 32 contained *miR-489* target sites, as predicted by the TargetScan database. More recently, we quickly and successfully screened miRNA target genes using microarray methods (Chiyomaru *et al*, 2010; Kano *et al*, 2010). Tumour-suppressive miRNAs usually prevent tumour development by inhibiting the activity of oncogenes (Esquela-Kerscher and Slack, 2006; Hammond, 2006; Zhang *et al*, 2007). Therefore, we expected that target genes of *miR-489* would have oncogenic functions. One of the 53 candidates, *PTPN11*, is a cytoplasmic PTP that contains two Src Homology 2 domains. These PTPs are generally negative regulators because of their ability to oppose the effects of protein tyrosine kinases. Our data demonstrate that *PTPN11* has an oncogenic role and is directly regulated by *miR-489* in HSCC cells.

The *PTPN11* gene is unusual in that it promotes the activation of RAS–MAPK signalling pathway in response to various growth factors and cytokines (Mohi and Neel, 2007; Matozaki *et al*, 2009). Interestingly, germline *PTPN11* mutations have been identified in patients with Noonan syndrome, juvenile myelomonocytic leukaemia, and paediatric acute leukaemia (Aoki *et al*, 2008). Mutation of *PTPN11* in Noonan syndrome and leukaemic cells resulted in gain-of-function enhanced phosphatase activity. Molecular and genetic studies have also shown that PTPN11 mediates cell signalling by epidermal growth factor (EGF), hepatocyte growth factor, and interleukin-6; specifically, PTPN11 has a role in the activation of ERK1/2 MAP kinase by EGF (Chen *et al*, 2006). The EGF signalling pathway is involved in a variety of cellular responses including cell growth and proliferation, and monoclonal antibodies and small-molecule inhibitors have been developed to inhibit EGF receptor (EGFR) pathways. These pathways, which include RAS–MAPK signalling, have been extensively studied in HNSCC, and seem to have a critical role in the survival and proliferation of cancer cells (Kalyankrishna and Grandis, 2006) and EGFR is overexpressed in more than 50% of HSCC specimens (Frank *et al*, 1993). Our data suggest that the silencing of *miR-489* expression, and subsequent overexpression of *PTPN11*, leads to abnormal EGFR signalling. Future studies will clarify the mechanism by which deregulation of EGFR signalling networks contributes to HSCC carcinogenesis.

This study is to identify tumour-suppressive miRNAs based on clinical HSCC miRNA expression signature. We have specifically identified a tumour-suppressive miRNA (*miR-489)* and found its direct target (*PTPN11*). Disruption of this interaction may lead to the deregulation of *miR-489-PTPN11* signalling in HSCC. The possibility of exploiting the therapeutic implications of these findings for future treatment of HSCC should be explored in future studies.

## Figures and Tables

**Table 1:** HSCC patients' characteristics for miRNA screening test

**Patient**		**Age**		**TNM stage**		
**number**	**Gender**	**(years)**	**Differentiation**	**T**	**N**	**M**
1	M	58	Well	3	2c	0
2	M	71	Moderate	1	0	0
3	M	60	Moderate	3	2c	0
4	M	69	Moderate	3	2c	0
5	M	60	Moderate	2	2c	0
6	F	74	Moderate	4a	2b	0
7	M	57	Moderate	4a	2c	0
8	M	62	Moderate	2	1	0
9	F	52	Well	4a	2b	0
10	M	56	Moderate	4a	2b	0

Abbreviations: HSCC=hypopharyngeal squamous cell carcinoma; miRNA=microRNA.

**Table 2:** (a) Upregulated miRNAs in HSCC and (b) downregulated miRNAs in HSCC

		**Normalized ratio**		
**Gene/miRNA**	**Fold change**	**Non-cancer**	**Cancer**	***P*-value**
(**A**)				
miR-517c	24.862	0.1511	3.7568	3.66E−05
miR-196a	10.073	0.7187	7.2388	1.40E−02
miR-7	9.301	0.5490	5.1059	1.80E−04
miR-196b	6.698	0.4192	2.8074	8.24E−04
miR-650	4.924	0.7011	3.4519	1.81E−02
miR-18a	3.518	0.6705	2.3590	2.76E−03
miR-452	3.478	0.7385	2.5683	2.93E−02
miR-183	3.063	0.6892	2.1110	2.93E−02
miR-432	3.027	0.5053	1.5296	2.38E−02
miR-301a	2.822	0.7331	2.0691	1.37E−02
miR-21	2.675	0.6324	1.6920	2.76E−03
				
(**B**)				
miR-1	0.007	59.2640	0.4360	2.40E−02
miR-375	0.033	4.0344	0.1322	7.25E−05
miR-139-5p	0.092	4.3646	0.4012	1.52E−04
miR-504	0.147	2.5714	0.3793	1.20E−02
miR-125b	0.232	2.2884	0.5314	7.15E−04
miR-199b	0.268	1.5739	0.4217	6.53E−03
miR-100	0.274	1.7713	0.4861	2.76E−03
miR-497	0.278	2.0062	0.5575	7.15E−04
let-7c	0.282	1.8374	0.5182	3.66E−03
miR-30a^*^	0.318	1.6777	0.5330	1.20E−02
miR-218	0.322	1.5021	0.4835	1.27E−02
miR-10b	0.328	1.9344	0.6353	5.89E−03
miR-126^*^	0.341	2.2839	0.7788	8.91E−03
miR-378	0.342	2.0853	0.7125	2.93E−02
miR-328	0.349	1.5447	0.5394	1.25E−03
miR-204	0.356	2.1216	0.7556	3.36E−02
miR-143	0.365	1.5665	0.5710	5.89E−03
miR-126	0.372	1.6993	0.6324	5.64E−04
miR-99a	0.374	1.3994	0.5229	2.93E−02
miR-195	0.393	1.7477	0.6864	1.29E−03
miR-489	0.404	1.6276	0.6572	9.07E−03
miR-203	0.446	1.4617	0.6512	3.36E−02
miR-140-5p	0.470	1.3766	0.6476	5.64E−04
miR-29a	0.484	1.4544	0.7046	5.23E−03
miR-26a	0.490	1.5074	0.7379	5.89E−03
miR-214	0.490	1.3913	0.6815	3.70E−02
miR-30a	0.546	1.3110	0.7162	5.89E−03
miR-26b	0.550	1.2776	0.7030	1.27E−02
miR-30e^*^	0.571	1.3696	0.7814	4.71E−02
miR-30b	0.610	1.3094	0.7984	1.27E−02
let-7b	0.618	1.3212	0.8162	3.95E−02

Abbreviations: HSCC=hypopharyngeal squamous cell carcinoma; miRNA=microRNA.

**Table 3:** Downregulated genes by miR-489 treatment in FaDu cells

**No.**	**Symbol**	**Gene Name**	**Gene ID**	**Location**	**Fold**	**Target sites**
1	*CTDSPL2*	CTD (carboxy-terminal domain, RNA polymerase II, polypeptide A) small phosphatase like 2	NM_016396	15q15.3	−3.59	3
2	*PTPN11*	Protein tyrosine phosphatase, non-receptor type 11 (Noonan syndrome 1)	NM_002834	12q24.13	−3.40	2
3	*GPR110*	G protein-coupled receptor 110	NM_025048	6p12.3	−3.05	—
4	*CLIP4*	CAP-GLY domain containing linker protein family, member 4	NM_024692	2p23.2	−2.87	1
5	*VGF*	VGF nerve growth factor inducible	NM_003378	7q22.1	−2.85	—
6	*CD244*	CD244 molecule, natural killer cell receptor 2B4	NM_016382	1q23.3	−2.72	—
7	*SUZ12*	Suppressor of zeste 12 homologue (*Drosophila*)	NM_015355	17q11.2	−2.68	3
8	*LIN28B*	Lin-28 homologue B (*Caenorhabditis elegans*)	NM_001004317	6q21	−2.68	1
9	*AP1S1*	Adaptor-related protein complex 1, sigma 1 subunit	NM_001283	7q22.1	−2.62	1
10	*NF2*	Neurofibromin 2 (merlin)	NM_181831	22q12.2	−2.55	1
11	*AP1M2*	Adaptor-related protein complex 1, mu 2 subunit	NM_005498	19p13.2	−2.54	1
12	*A2ML1*	Alpha-2-macroglobulin-like 1	NM_144670	12p13.31	−2.52	—
13	*CRIPT*	Cysteine-rich PDZ-binding protein	NM_014171	2p21	−2.51	1
14	*EGR1*	Early growth response 1	NM_001964	5q31.2	−2.51	—
15	*CYP1B1*	Cytochrome P450, family 1, subfamily B, polypeptide 1	NM_000104	2p22.2	−2.49	2
16	*NAP1L1*	Nucleosome assembly protein 1-like 1	NM_139207	12q21.2	−2.48	—
17	*AHNAK*	AHNAK nucleoprotein	NM_001620	11q12.3	−2.48	—
18	*FAM26E*	Family with sequence similarity 26, member E	NM_153711	6q22.1	−2.43	1
19	*RAVER2*	Ribonucleoprotein, PTB-binding 2	NM_018211	1p31.3	−2.42	—
20	*RASL10A*	RAS-like, family 10, member A	NM_001007279	22q12.2	−2.40	—
21	*C14orf147*	Chromosome 14 open reading frame 147	NM_138288	14q13.1	−2.38	1
22	*C14orf143*	Chromosome 14 open reading frame 143	NM_145231	14q32.11	−2.37	1
23	*HTR2B*	5-hydroxytryptamine (serotonin) receptor 2B	NM_000867	2q37.1	−2.37	1
24	*MYLK*	Myosin light chain kinase	NM_053025	3q21.1	−2.33	—
25	*TFAP4*	Transcription factor AP-4 (activating enhancer-binding protein 4)	NM_003223	16p13.3	−2.33	1
26	*MYO3B*	Myosin IIIB	NM_138995	2q31.1	−2.32	1
27	*OSTM1*	Osteopetrosis-associated transmembrane protein 1	NM_014028	6q21	−2.32	1
28	*MARCKS*	Myristoylated alanine-rich protein kinase C substrate	NM_002356	6q22.1	−2.29	2
29	*KCTD4*	Potassium channel tetramerisation domain-containing 4	NM_198404	13q14.12	-2.26	1
30	*GCLC*	Glutamate-cysteine ligase, catalytic subunit	NM_001498	6p12.1	−2.26	—
31	*ERRFI1*	ERBB receptor feedback inhibitor 1	NM_018948	1p36.23	−2.26	1
32	*MDH1*	Malate dehydrogenase 1, NAD (soluble)	NM_005917	2p15	−2.26	—
33	*IL15*	Interleukin 15	NM_172174	4q31.21	−2.26	—
34	*ZCCHC5*	Zinc finger, CCHC domain-containing 5	NM_152694	Xq21.1	−2.26	—
35	*GRB10*	Growth factor receptor-bound protein 10	NM_001001555	7p12.2	−2.25	2
36	*KLHL5*	Kelch-like 5 (*Drosophila*)	NM_015990	4p14	−2.21	1
37	*BLID*	BH3-like motif containing, cell death inducer	NM_001001786	11q24.1	−2.20	—
38	*CFL2*	Cofilin 2 (muscle)	NM_021914	14q13.2	−2.19	3
39	*SLC24A1*	Solute carrier family 24 (sodium/potassium/calcium exchanger), member 1	NM_004727	15q22.31	−2.17	1
40	*CDIPT*	CDP-diacylglycerol–inositol 3-phosphatidyltransferase (phosphatidylinositol synthase)	NM_006319	16p11.2	−2.14	1
41	*RTP4*	Receptor (chemosensory) transporter protein 4	NM_022147	3q27.3	−2.14	—
42	*ATP1B3*	ATPase, Na+/K+ transporting, beta 3 polypeptide	NM_001679	3q23	−2.14	1
43	*NCOA3*	Nuclear receptor coactivator 3	NM_181659	20q13.12	−2.14	2
44	*CDK6*	Cyclin-dependent kinase 6	NM_001259	7q21.2	−2.12	1
45	*RP11-11C5.2*	Similar to RIKEN cDNA 2410129H14	NM_001071775	13q22.1	−2.11	2
46	*UNQ9438*	TIMM9	NM_207377	14q23.1	−2.08	—
47	*MAGEH1*	Melanoma antigen family H, 1	NM_014061	Xq11.21	−2.07	—
48	*HPS3*	Hermansky–Pudlak syndrome 3	NM_032383	3q24	−2.05	1
49	*RNF149*	Ring finger protein 149	NM_173647	2q11.2	−2.04	—
50	*NUPL1*	Nucleoporin-like 1	NM_014089	13q12.13	−2.03	—
51	*SLC25A40*	Solute carrier family 25, member 40	NM_018843	7q21.12	−2.03	—
52	*ZCCHC4*	Zinc finger, CCHC domain-containing 4	NM_024936	4p15.2	−2.02	1
53	*TMEM64*	Transmembrane protein 64	NM_001008495	8q21.3	−2.01	1
